# A Comparative Evaluation of the Effects of Saroglitazar and Pioglitazone on Hepatic and Metabolic Parameters in Type 2 Diabetes Mellitus With Metabolic Dysfunction-Associated Steatotic Liver Disease (MASLD)

**DOI:** 10.7759/cureus.100002

**Published:** 2025-12-24

**Authors:** Dayanidhi Meher, Sambit Das, Arun Choudhury, Devadarshini Sahoo, Sandeep K Sahu, Vishal Agarwal, Binod Prusty, Bijay Ketan Das, Amogh S Chappalagavi, Sheenam Gupta, Vidhya Sreekumar

**Affiliations:** 1 Endocrinology, Kalinga Institute of Medical Sciences, Bhubaneswar, IND; 2 Endocrinology, Diabetes and Metabolism, Kalinga Institute of Medical Sciences, Bhubaneswar, IND; 3 General Medicine, Government Medical College and Hospital, Chandigarh, IND; 4 Medical Affairs, Micro Labs Private Limited, Bangalore, IND

**Keywords:** hepatic fibrosis, masld, pioglitazone, saroglitazar, type 2 diabetes mellitus

## Abstract

Background and objective

Type 2 diabetes mellitus (T2DM) is frequently accompanied by metabolic dysfunction-associated steatotic liver disease (MASLD), compounding morbidity and mortality risks. Pharmacologic strategies that directly target hepatic steatosis and fibrosis in this population remain limited. Saroglitazar, a dual PPARα/γ agonist, and pioglitazone, a proliferator-activated receptor gamma (PPAR-γ) agonist, may offer potential benefits. This study aimed to compare their effects on hepatic, glycemic, and metabolic parameters in patients with T2DM and MASLD.

Methods

In this study, 96 adults with T2DM and MASLD were randomized to receive either pioglitazone 15 mg or saroglitazar 4 mg daily for 12 months. Primary outcomes included changes in the non-alcoholic fatty liver disease (NAFLD) fibrosis score (NFS), liver stiffness measurement (LSM), and controlled attenuation parameter (CAP). Secondary outcomes assessed fasting plasma glucose (FPG), postprandial glucose (PPPG), HbA1c, lipid profile, triglyceride-glucose (TyG) index, and BMI. Assessments were performed at baseline and at three, six, and 12 months. Data were analyzed using paired and unpaired t-tests.

Results

Saroglitazar produced a significant reduction in NFS (-0.57, p = 0.026) and a non-significant decrease in LSM (-1.1 kPa). CAP declined by 18.6 dB/m (p = 0.059). HbA1c dropped by 0.81% (p<0.0001) with saroglitazar, surpassing pioglitazone’s 0.31% reduction. Both groups showed modest improvements in lipid levels and stable TyG indices. Saroglitazar led to a significant BMI reduction (-3.32 kg/m², p = 0.006); pioglitazone showed no BMI change. Both treatments were well tolerated, with no major adverse events reported.

Conclusions

Saroglitazar demonstrated superior benefits over pioglitazone in improving hepatic steatosis and fibrosis, glycemic control, and BMI in patients with T2DM and MASLD, while maintaining a favorable safety profile.

## Introduction

Type 2 diabetes mellitus (T2DM) is a global public health challenge with an increasing prevalence, which is attributed to sedentary lifestyles, poor dietary habits, and urbanization. India, often referred to as the “Diabetes Capital of the World,” is projected to have more than 80 million individuals living with diabetes by 2030 [1]. The escalating burden of T2DM is closely linked to metabolic liver disorders, particularly metabolic dysfunction-associated steatotic liver disease (MASLD), due to shared pathogenic mechanisms such as insulin resistance and dyslipidemia. In fact, MASLD is considered a tissue-specific form of insulin resistance in the liver. The prevalence of MASLD among individuals with T2DM is alarmingly high, with recent global meta-analyses estimating a pooled prevalence of approximately 65 to 70%. This reflects a rising trend from earlier estimates, such as 55.48% reported in 2019, increasing to 65.33% in more recent reviews [2].

Regional variations are pronounced, with the highest prevalence reported in Turkey (94.35%) and Eastern Europe (80.62%), while lower rates are seen in Latin America (51.35%) and parts of Asia. In India, MASLD affects an estimated 55 to 65% of adults with T2DM, based on both hospital-based studies and large cross-sectional surveys [3]. Some urban Indian cohorts report prevalence as high as 65.9%, highlighting the disease’s substantial burden in this high-risk population. The rising prevalence mirrors global metabolic trends, including increasing obesity, insulin resistance, and sedentary behavior, as well as increased caloric intake, which contribute to the progression of MASLD to more severe forms, including metabolic dysfunction-associated steatohepatitis (MASH) and advanced liver fibrosis, including liver cirrhosis [4].

Lifestyle modification remains the cornerstone of MASLD management, with evidence indicating resolution of steatosis in up to 90% of patients and improvement in liver fibrosis in approximately 45% of cases [5,6]. However, pharmacological interventions are increasingly necessary, particularly for individuals with co-existing T2DM and dyslipidemia, for whom lifestyle measures alone may not be sufficient. Several drug classes have demonstrated potential therapeutic benefits. Pioglitazone, a peroxisome proliferator-activated receptor gamma (PPAR-γ) agonist, enhances insulin sensitivity and reduces hepatic triglyceride accumulation, though its use is limited by adverse effects such as fluid retention and an increased risk of heart failure, and a higher incidence of fractures in the elderly population. Glucagon-like peptide-1 receptor agonists (GLP-1 RAs), including liraglutide and semaglutide, have shown efficacy in inducing weight loss and improving hepatic steatosis. Sodium-glucose co-transporter-2 (SGLT2) inhibitors also offer cardiometabolic benefits and reduce hepatic fat content, although robust histological evidence remains limited [7-9].

Saroglitazar is a novel dual PPAR α/γ agonist with predominant PPAR-α activity and moderate PPAR-γ activity [10]. It was approved in India in 2013 for the management of diabetic dyslipidemia and hypertriglyceridemia in patients with T2DM who were inadequately controlled on statins and fibrates, and subsequently, in 2020, received approval for use in type 2 diabetes and non-cirrhotic MASH [11,12]. Saroglitazar is currently the only dual PPAR α/γ agonist approved for clinical use worldwide. It has demonstrated favourable effects on both lipid and glycemic parameters, with a low incidence of adverse effects such as weight gain or peripheral edema [13].

Given the increasing burden of MASLD in diabetic patients and the lack of consensus on optimal pharmacotherapy, this study was undertaken to evaluate and compare the effects of saroglitazar and pioglitazone on liver steatosis, hepatic fibrosis, glycemic control, and lipid parameters in patients with T2DM and MASLD. By assessing changes in MASLD non-alcoholic fatty liver disease (NAFLD) fibrosis score (NFS), FibroScan parameters, and metabolic markers, this study aims to provide insights into the therapeutic potential of these agents and address an important clinical need in the management of diabetes-associated MASLD.

## Materials and methods

Study design and setting

This was a prospective, hospital-based comparative study conducted over a period of two years, from October 2018 to September 2020. The study was carried out in the Department of Pharmacology in collaboration with the Department of Endocrinology, Kalinga Institute of Medical Sciences (KIMS), Bhubaneswar, Odisha.

Study objectives

The primary objective of this study was to compare the NFS between patients with T2DM receiving saroglitazar and those receiving pioglitazone over a 12-month treatment period. Secondary objectives included the assessment and comparison of radiological changes in hepatic steatosis using FibroScan parameters, such as the controlled attenuation parameter (CAP) score and liver stiffness measurement (LSM). Additionally, the study aimed to evaluate changes in glycemic control through fasting plasma glucose (FPG), postprandial plasma glucose (PPPG), and HbA1c levels, as well as changes in lipid parameters, including total cholesterol, low/high-density lipoprotein (LDL/HDL) cholesterol, and triglycerides. Safety was also assessed by documenting and comparing adverse drug reactions (ADRs) associated with each treatment arm.

Study participants

**Figure 1:**
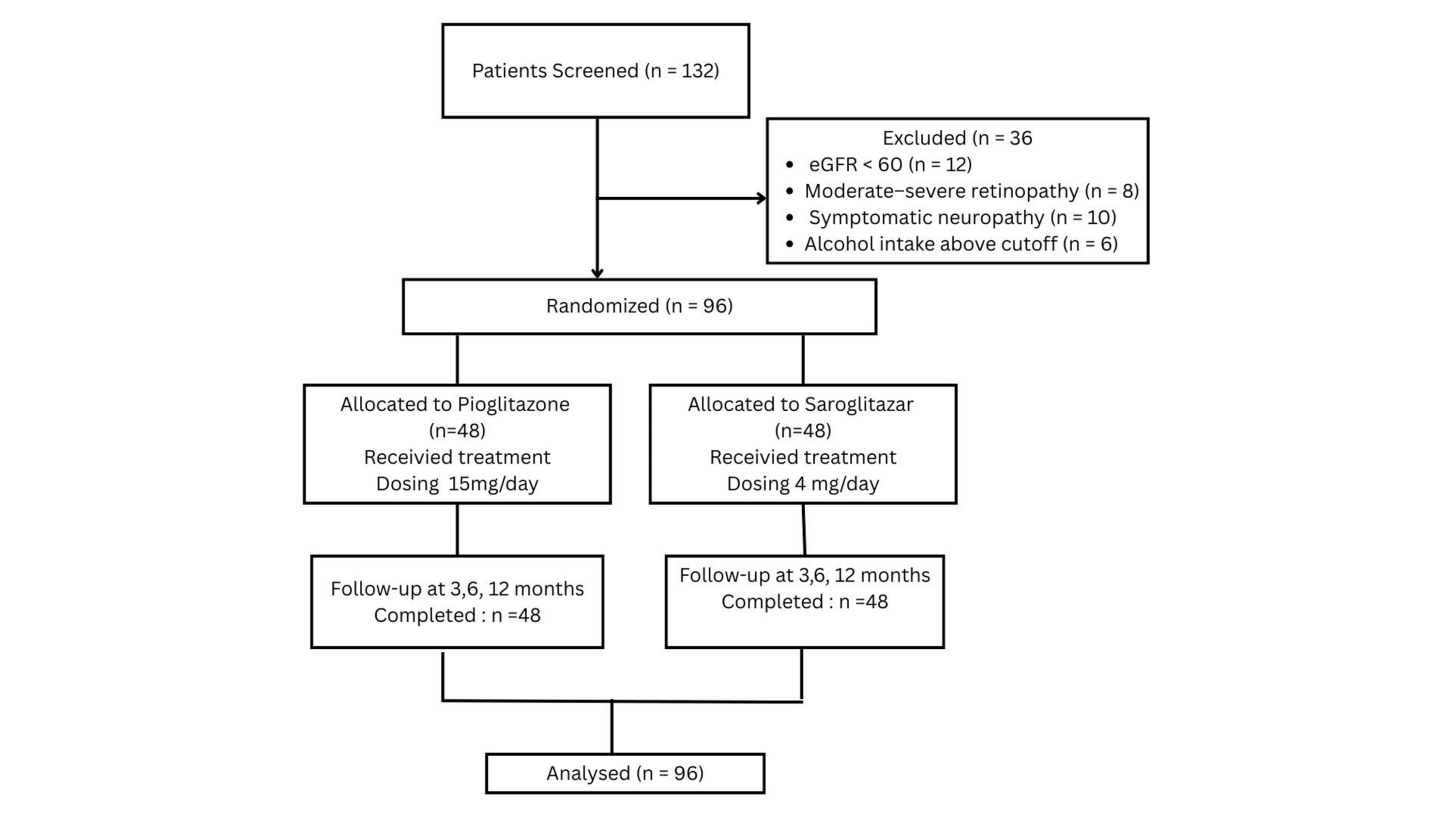
CONSORT flow diagram depicting patient screening CONSORT: Consolidated Standards of Reporting Trials; eGFR: estimated glomerular filtration rate

A total of 96 adults with T2DM and MASLD were screened and enrolled. Participants were identified through outpatient and inpatient services. Screening included clinical evaluation, laboratory testing, and FibroScan assessment. MASLD diagnosis was confirmed using a CAP >260 dB/m and NFS >0.67, following established diagnostic thresholds (Figure 1). At baseline, all participants were receiving stable background antidiabetic therapy, most commonly metformin with or without sulfonylureas or dipeptidyl peptidase 4 (DPP-4) inhibitors. Background medications were continued unchanged throughout the study period unless rescue therapy criteria were met.

Participants were eligible for inclusion if they were adults above 18 years of age of either sex, with a documented history of T2DM of less than five years’ duration. Additional inclusion requirements were a BMI greater than 30 kg/m², FPG exceeding 126 mg/dL, PPPG greater than 200 mg/dL, and an HbA1c level of at least 6.5%. Dyslipidemia was required and defined as total cholesterol more than 200 mg/dL, LDL cholesterol above 100 mg/dL, HDL cholesterol below 40 mg/dL in men or below 50 mg/dL in women, and triglycerides above 150 mg/dL. Eligible participants also demonstrated mildly elevated liver enzymes (aspartate aminotransferase (AST) >38 U/L and alanine aminotransferase (ALT) >40 U/L) with preserved hepatic and renal function, indicated by serum albumin below 50 g/L, platelet count above 145×10³/mm³, serum creatinine less than 1.2 mg/dL in men and less than 0.9 mg/dL in women, and an estimated glomerular filtration rate (eGFR) of at least 60 mL/min/1.73 m² (CKD-EPI equation).

Participants were excluded if they reported alcohol intake exceeding 210 mL per week for men or 140 mL per week for women. Individuals with advanced diabetic complications were also excluded, including those with moderate non-proliferative diabetic retinopathy (NPDR) or worse, macroalbuminuria or eGFR below 60 mL/min/1.73 m² indicating nephropathy, or symptomatic peripheral neuropathy with clinical or electrophysiological evidence of distal sensorimotor polyneuropathy. Additional exclusion criteria comprised a history of coronary artery disease, autoimmune liver disease, viral hepatitis, or any malignancy. Pregnant or lactating women and individuals receiving hepatotoxic medications were excluded from participation. The rescue criteria were as follows: during study follow-up, if a participant’s HbA1c levels were between 7.0 and 7.5%, additional anti-diabetic medication was administered at the investigator’s discretion.

Sample size calculation

Sample size estimation was based on detecting a clinically meaningful difference in NFS between groups, informed by the effect size from the study by Goyal et al. (2020). Using SD = 7.29 and expected difference (d) = 5.3, the following formula was applied:

Where:

Zα = 1.96 (95% confidence)

Z1−β = 1.28 (90% power)

β = 10%

SD and d taken from previous MASLD pharmacotherapy data

The calculated sample size was 40 per group. Allowing for a 20% attrition rate, the final sample size was 48 participants per arm (total N = 96).

Randomization and intervention

Eligible participants were randomly allocated in a 1:1 ratio to receive either pioglitazone or saroglitazar. Block randomization (block size: four) was performed using a computer-generated random sequence by an independent statistician not involved in outcome assessment. Allocation concealment was ensured using opaque sealed envelopes opened sequentially. Group I received pioglitazone 15 mg once daily, and Group II received saroglitazar 4 mg once daily, both administered before breakfast for 12 months. Participants previously on other oral antidiabetic drugs were transitioned to the assigned study medication over a two-month titration period. Rescue therapy criteria were predefined. During follow-up, if a participant’s HbA1c increased to a range between 7.0% and 7.5%, indicating inadequate glycemic control, additional antidiabetic medication was introduced at the investigator’s discretion. No other glucose-lowering agents were permitted unless this rescue threshold was met.

Assessment and follow-up

Baseline evaluation included anthropometry, laboratory work-up, and FibroScan® (VCTE) assessment. Liver fat content was quantified using the CAP, and liver stiffness using LSM. These parameters were selected because CAP correlates with steatosis grade and LSM correlates with fibrosis severity in MASLD. NFS was calculated using age, BMI, glycemia, platelet count, albumin, and AST/ALT ratio as per the original validated formula. Follow-up visits occurred at three, six, and 12 months. At each visit, glycemic and lipid parameters, liver enzymes, renal indices, and ADRs were recorded. All participants received standard lifestyle counseling on diet optimization, weight reduction, and physical activity. However, adherence to lifestyle measures was not monitored or recorded, which is acknowledged as a limitation. FibroScan operators were not blinded to treatment allocation. Blinding was not implemented because FibroScan generates automated quantitative measurements, and the primary endpoint (NFS) is laboratory-based and not operator-dependent. A repeat FibroScan was performed at 12 months to quantify changes in steatosis (CAP) and stiffness (LSM).

Statistical analysis

Data analysis was performed using Microsoft Excel and IBM SPSS Statistics version 20.0 (IBM Corp., Armonk, NY). Continuous variables were presented as mean ± standard deviation (SD), while categorical variables were expressed as frequencies and percentages. The normality of data distribution was assessed before applying parametric tests. Intra-group comparisons (baseline vs. follow-up) for continuous variables were conducted using paired t-tests. Inter-group comparisons between the saroglitazar and pioglitazone groups were carried out using unpaired t-tests. Categorical variables were analyzed using the chi-square test where appropriate. A p-value <0.05 was considered statistically significant for all analyses.

Ethical considerations

The study was conducted in accordance with the principles outlined in the Declaration of Helsinki and Good Clinical Practice (GCP) guidelines. Before the initiation of the study, ethical clearance was obtained from the Institutional Ethics Committee (IEC) and the Institutional Research Committee of Kalinga Institute of Medical Sciences (KIMS), Bhubaneswar (registration No. KIIMS/KIIT/IEC/108/2018). Informed written consent was obtained from all participants before their enrolment in the study.

## Results

At baseline, participants in both groups were comparable in terms of age, gender distribution, BMI, and most metabolic parameters. However, the saroglitazar group had significantly higher FPG, HbA1c, liver enzymes (AST and ALT), LSM scores, CAP scores, and NFS, suggesting a more advanced metabolic and hepatic disease profile at study entry. LDL cholesterol levels were also significantly lower in the saroglitazar group. These baseline differences between the groups are summarized in Table 1.

**Table 1:** Baseline demographic and clinical parameters BCA: body composition analyzer; FPG: fasting plasma glucose; PPPG: postprandial plasma glucose; HbA1c: glycated hemoglobin; AST: aspartate aminotransferase; ALT: alanine aminotransferase; LSM: liver stiffness measurement; CAP: controlled attenuation parameter; NFS: non-alcoholic fatty liver disease (NAFLD) fibrosis score; LDL: low-density lipoprotein; HDL: high-density lipoprotein; TyG: triglyceride-glucose index; SD: standard deviation

Parameter	Pioglitazone (n = 48)	Saroglitazar (n = 48)	P-value
Age, year, mean ± SD	56.23 ± 8.48	52.88 ± 9.39	0.087
Gender, n (%)			
Male	27 (69.23%)	30 (62.5%)	0.66
Female	12 (30.77%)	18 (37.5%)	
BCA parameters, mean ± SD			
Weight, kg	73.92 ± 12.19	78.81 ± 16.03	0.12
Height, cm	159.14 ± 9.43	160.69 ± 11.09	0.49
BMI, kg/m^2^	29.37 ± 5.69	30.73 ± 6.22	0.3
Glycemic profile, mean ± SD			
FPG, mg/dL	155.1 ± 47.08	186.15 ± 75	0.03
PPPG, mg/dL	205.95 ± 67.84	201.88 ± 85.09	0.81
HbA1c, %	6.57 ± 1.28	7.38 ± 1.17	0.003
Liver profile, , mean ± SD			
AST, U/L	27.62 ± 9.31	33.76 ± 13.73	0.02
ALT, U/L	32.9 ± 15.67	46.54 ± 25.56	0.004
LSM, kPa	5 ± 0.91	8 ± 4.9	0.0004
CAP, dB/m	298 ± 57.94	321.4 ± 49.32	0.04
NFS	1.06 ± 1.85	1.71 ± 1.23	0.05
Lipid profile, mean ± SD			
Cholesterol, mg/dL	169.8 ± 41.09	165.83 ± 46.47	0.68
Triglyceride, mg/dL	205.56 ± 162.96	181.31 ± 82.98	0.37
LDL, mg/dL	102.82 ± 32.96	87.21 ± 36.15	0.04
HDL, mg/dL	40.54 ± 11.69	40.1 ± 8.84	0.84
TyG index	5.07 ± 0.33	5.12 ± 0.3	0.46
Kidney profile, mean ± SD			
Serum albumin, g/dL	5.15 ± 3.08	4.45 ± 0.52	0.12
Serum creatinine, mg/dL	0.89 ± 0.32	1.1 ± 0.89	0.15

Effect on liver fibrosis

Over the 12-month follow-up, saroglitazar use led to a reduction in liver stiffness by 1.1 kPa as measured by LSM, whereas no change was observed in the pioglitazone group. Similarly, hepatic fat content assessed using CAP scores declined by 18.6 dB/m with saroglitazar and 10.4 dB/m with pioglitazone. The most clinically relevant improvement was observed in the NFS, which decreased by 0.57 points in the saroglitazar group over 12 months (p = 0.026). Intra-group analysis demonstrated a significant reduction in NFS within the saroglitazar group (p = 0.026), whereas changes in FibroScan and CAP in both groups were not statistically significant, although the p-value for CAP in the saroglitazar group was 0.059 (Table 2).

**Table 2:** Comparison of liver fibrosis parameters between the groups at baseline and 12-month follow-up Between-group comparisons were performed using the independent t-test, while within-group (intra-group) comparisons from baseline to 12 months were analyzed using the paired t-test LSM: liver stiffness measurement; CAP: controlled attenuation parameter; NFS: non-alcoholic fatty liver disease (NAFLD) fibrosis score; SD: standard deviation

Parameter	Time point	Pioglitazone	Saroglitazar	P-value
LSM, kPa	Baseline, mean ± SD	5 ± 0.91	8 ± 4.9	0.0004
	12 months, mean ± SD	5 ± 0.71	6.9 ± 3.6	0.0018
	P-value (paired t-test)	0.83	0.21	-
CAP, dB/m	Baseline, mean ± SD	298 ± 57.94	321.4 ± 49.32	0.04
	12 months, mean ± SD	287.64 ± 55.17	302.81 ± 45.77	0.16
	P-value (paired t-test)	0.42	0.059	-
NFS	Baseline, mean ± SD	1.06 ± 1.85	1.71 ± 1.23	0.05
	12 months, mean ± SD	1.08 ± 1.51	1.14 ± 1.21	0.83
	P-value (paired t-test)	0.97	0.026	

Effect on glycemic parameters

Change in FPG levels declined by 26.92 mg/dL in the saroglitazar group and by 18.97 mg/dL in the pioglitazone group, though the inter-group difference was not statistically significant at the end of 12-month follow-up (p = 0.064). PPPG also decreased in both groups, with a slightly greater reduction observed in the pioglitazone group (-28.79 mg/dL vs. -19.5 mg/dL), but the difference remained non-significant at 12 months (p = 0.72). The most notable changes were observed in HbA1c levels, which declined by 0.81% in the saroglitazar group and 0.31% in the pioglitazone group over six months, although values partially rebounded by 12 months, thereby narrowing the inter-group difference (p = 0.1) (Table 3).

**Table 3:** Comparison of inter-group glycemic parameters between groups at baseline and follow-up visits Between-group comparisons at each time point were analyzed using the independent t-test FPG: fasting plasma glucose; PPPG: postprandial plasma glucose; HbA1c: glycated hemoglobin; SD: standard deviation

Parameter	Time point	Pioglitazone, mean ± SD	Saroglitazar, mean ± SD	P-value
FPG, mg/dL	Baseline	155.1 ± 47.08	186.15 ± 75	0.03
	3 months	143.13 ± 40.06	172.17 ± 72.98	0.03
	6 months	139.77 ± 39.76	164.44 ± 69.9	0.053
	12 months	136.13 ± 41.85	159.23 ± 66.08	0.064
PPPG, mg/dL	Baseline	205.95 ± 67.84	201.88 ± 85.09	0.81
	3 months	179.87 ± 57.94	196.42 ± 83.86	0.03
	6 months	178.1 ± 51.91	187.69 ± 79.94	0.52
	12 months	177.16 ± 52.68	182.38 ± 75.81	0.72
HbA1c, %	Baseline	6.57 ± 1.28	7.38 ± 1.17	0.003
	3 months	6.33 ± 1.19	6.98 ± 0.90	0.0046
	6 months	6.09 ± 1.07	6.52 ± 0.85	0.041
	12 months	6.26 ± 1.08	6.57 ± 0.64	0.10

Intra-group analysis showed a statistically significant reduction in HbA1c within the saroglitazar group over time (p<0.0001), while changes in FPG and PPPG in both groups were not statistically significant (Table 4).

**Table 4:** Intra-group changes in glycemic parameters over 12 months Within-group changes from baseline to 12 months were analyzed using repeated-measures ANOVA FPG: fasting plasma glucose; PPPG: postprandial plasma glucose; HbA1c: glycated hemoglobin; SD: standard deviation; ANOVA: analysis of variance

Parameter	Group	Baseline, mean ± SD	12 months, mean ± SD	ANOVA p-value
FPG, mg/dL	Pioglitazone	155.1 ± 47.08	136.13 ± 41.85	0.23
	Saroglitazar	186.15 ± 75	159.23 ± 66.08	0.28
PPPG, mg/dL	Pioglitazone	205.95 ± 67.84	177.16 ± 52.68	0.089
	Saroglitazar	201.88 ± 85.09	182.38 ± 75.81	0.65
HbA1c, %	Pioglitazone	6.57 ± 1.28	6.26 ± 1.08	0.33
	Saroglitazar	7.38 ± 1.17	6.57 ± 0.64	<0.0001

Effect on liver and renal function parameters

Over the 12 months, AST levels showed a mild reduction in both groups, with a change of -5.56 U/L in the saroglitazar group and -0.3 U/L in the pioglitazone group. However, these changes were not statistically significant by the end of the study (p = 0.66). ALT levels followed a similar trend, with a reduction of -9.48 U/L in the saroglitazar group and -0.98 U/L in the pioglitazone group, with no significant between-group difference at 12 months (p = 0.2) (Table 5). Intra-group analysis showed a significant reduction in AST over time within the saroglitazar group (p = 0.05), while changes in the pioglitazone group remained non-significant (Table 6). Regarding renal parameters, serum albumin levels significantly decreased within the saroglitazar group over 12 months (p<0.0001), with a mean reduction of 0.26 g/dL, while no significant change was observed in the pioglitazone group (p = 0.18). Serum creatinine remained stable in both groups throughout the study period.

**Table 5:** Comparison of liver enzymes and renal function between groups at baseline and follow-up visits Between-group comparisons at each time point were analyzed using the independent t-test AST: aspartate aminotransferase; ALT: alanine aminotransferase; SD: standard deviation

Parameter	Time point	Pioglitazone, mean ± SD	Saroglitazar, mean ± SD	P-value
AST, U/L	Baseline	27.62 ± 9.31	33.76 ± 13.73	0.02
	3 months	26.51 ± 9.31	30.69 ± 13.37	0.10
	6 months	24.77 ± 8.34	27.17 ± 12.61	0.31
	12 months	27.32 ± 6.25	28.20 ± 10.95	0.66
ALT, U/L	Baseline	32.90 ± 15.67	46.54 ± 25.56	0.004
	3 months	32.74 ± 16.57	42.99 ± 25.43	0.033
	6 months	30.41 ± 15.92	38.56 ± 24.98	0.08
	12 months	31.92 ± 13.30	37.06 ± 21.50	0.20
Serum albumin, g/dL	Baseline	5.15 ± 3.08	4.45 ± 0.52	0.12
	3 months	4.49 ± 0.98	4.04 ± 0.47	0.007
	6 months	4.33 ± 1.05	3.79 ± 0.47	0.002
	12 months	4.47 ± 1.10	4.19 ± 0.68	0.14
Serum creatinine, mg/dL	Baseline	0.89 ± 0.32	1.10 ± 0.89	0.15
	3 months	0.78 ± 0.34	0.92 ± 0.91	0.36
	6 months	0.83 ± 0.36	0.88 ± 0.77	0.71
	12 months	0.87 ± 0.32	0.85 ± 0.62	0.85

**Table 6:** Intra-group changes in liver and renal parameters over 12 months Within-group changes from baseline to 12 months were analyzed using repeated-measures ANOVA AST: aspartate aminotransferase; ALT: alanine aminotransferase; SD: standard deviation; ANOVA: analysis of variance

Parameter	Group	Baseline, mean ± SD	12 months	ANOVA p-value
AST, U/L	Pioglitazone	27.62 ± 9.31	27.32 ± 6.25	0.44
	Saroglitazar	33.76 ± 13.73	28.20 ± 10.95	0.05
ALT, U/L	Pioglitazone	32.90 ± 15.67	31.92 ± 13.30	0.89
	Saroglitazar	46.54 ± 25.56	37.06 ± 21.50	0.22
Serum albumin, g/dL	Pioglitazone	5.15 ± 3.08	4.47 ± 1.10	0.18
	Saroglitazar	4.45 ± 0.52	4.19 ± 0.68	<0.0001
Serum creatinine, mg/dL	Pioglitazone	0.89 ± 0.32	0.87 ± 0.32	0.54
	Saroglitazar	1.10 ± 0.89	0.85 ± 0.62	0.42

Effect on lipid profile

Over the 12 months, both groups showed mild reductions in total cholesterol and triglycerides, but none of the changes were statistically significant between or within groups (Table 7). LDL cholesterol, which was significantly lower in the saroglitazar group at baseline (p = 0.04), remained lower throughout follow-up, though the difference lost statistical significance over time. HDL levels showed a slight increase in both groups, with a mean rise of 1.44 mg/dL in the saroglitazar arm, but this too did not reach statistical significance (p = 0.087). Intra-group changes in LDL, HDL, and triglycerides remained non-significant across both treatment arms (Table 8). The triglyceride-glucose (TyG) index, a surrogate marker for insulin resistance, remained stable in both groups, with a minor reduction of 0.10 points in the saroglitazar group and 0.09 points in the pioglitazone group. No statistically significant differences were observed either between or within groups across any follow-up visits, suggesting minimal effect of either treatment on insulin resistance over 12 months.

**Table 7:** Comparison of lipid profile between groups at baseline and follow-up visits ^*^Statistically significant between-group difference at baseline (p<0.05) Between-group comparisons at each time point were analyzed using the independent t-test LDL: low-density lipoprotein; HDL: high-density lipoprotein; TyG: triglyceride-glucose; SD: standard deviation

Parameter	Time point	Pioglitazone, mean ± SD	Saroglitazar, mean ± SD
Cholesterol, mg/dL	Baseline	169.8 ± 41.09	165.83 ± 46.47
	3 months	164.79 ± 39.42	163.96 ± 43.05
	6 months	161.7 ± 38.99	162.38 ± 40.54
	12 months	160.81 ± 37.7	161.21 ± 37.75
Triglycerides, mg/dL	Baseline	205.56 ± 162.96	181.31 ± 82.98
	3 months	200.92 ± 163.72	179.4 ± 79.84
	6 months	199 ± 162.75	177.1 ± 76.47
	12 months	194.41 ± 158.7	175.1 ± 72.99
LDL, mg/dL	Baseline	102.82 ± 32.96 *	87.21 ± 36.15^*^
	3 months	104.66 ± 31.31	92.39 ± 34.44
	6 months	106.35 ± 28.05	96.98 ± 33.57
	12 months	111.11 ± 25.48	102.35 ± 31.32
HDL, mg/dL	Baseline	40.54 ± 11.69	40.1 ± 8.84
	3 months	39.86 ± 8.44	39.98 ± 6.91
	6 months	39.68 ± 5.72	41.15 ± 5.92
	12 months	40.41 ± 3.75	41.85 ± 3.89
TyG index	Baseline	5.07 ± 0.33	5.12 ± 0.30
	3 months	5.02 ± 0.33	5.08 ± 0.29
	6 months	5.00 ± 0.32	5.06 ± 0.27
	12 months	4.97 ± 0.38	5.04 ± 0.25

**Table 8:** Intra-group changes in lipid profile over 12 months Within-group changes from baseline to 12 months were analyzed using repeated-measures ANOVA LDL: low-density lipoprotein; HDL: high-density lipoprotein; TyG: triglyceride-glucose; SD: standard deviation; ANOVA: analysis of variance

Parameter	Group	Baseline, mean ± SD	12 months, mean ± SD	ANOVA p-value
Cholesterol, mg/dL	Pioglitazone	169.8 ± 41.09	160.81 ± 37.7	0.75
	Saroglitazar	165.83 ± 46.47	161.21 ± 37.75	0.95
Triglycerides, mg/dL	Pioglitazone	205.56 ± 162.96	194.41 ± 158.7	0.99
	Saroglitazar	181.31 ± 82.98	175.1 ± 72.99	0.98
LDL, mg/dL	Pioglitazone	102.82 ± 32.96	111.11 ± 25.48	0.65
	Saroglitazar	87.21 ± 36.15	102.35 ± 31.32	0.16
HDL, mg/dL	Pioglitazone	40.54 ± 11.69	40.41 ± 3.75	0.96
	Saroglitazar	40.1 ± 8.84	41.85 ± 3.89	0.46
TyG index	Pioglitazone	5.07 ± 0.33	4.97 ± 0.38	0.57
	Saroglitazar	5.12 ± 0.30	5.04 ± 0.25	0.52

Effect on BMI

Over the 12-month follow-up, BMI reduced by 3.32 kg/m² in the saroglitazar group compared to 0.18 kg/m² in the pioglitazone group (Table 9). The inter-group difference at 12 months did not reach statistical significance (p = 0.13); however, intra-group analysis demonstrated a statistically significant reduction in BMI within the saroglitazar group (p = 0.006), whereas the pioglitazone group showed no significant change (p = 0.89).

**Table 9:** Comparison of BMI changes in pioglitazone and saroglitazar groups at baseline and 12-month follow-up Between-group differences were analyzed using the independent t-test, and within-group (intra-group) changes from baseline to 12 months were analyzed using the paired t-test BMI: body mass index; SD: standard deviation

Time point	Pioglitazone, mean ± SD	Saroglitazar, mean ± SD	P-value (inter-group)
Baseline	29.37 ± 5.69	30.73 ± 6.22	0.30
12 months	29.19 ± 5.44	27.41 ± 5.37	0.13
P-value (intra-group)	0.89	0.006	-

## Discussion

In this prospective comparative study, the comparative effects of saroglitazar and pioglitazone over 12 months were evaluated in patients with T2DM and MASLD, focusing on hepatic, glycemic, and metabolic parameters. Saroglitazar therapy was associated with greater improvements in liver steatosis, hepatic fibrosis, glycemic control, and BMI compared to pioglitazone. Changes in lipid profile and insulin resistance markers were modest in both groups. Both agents demonstrated acceptable safety profiles without any major adverse events during the study period. Importantly, the primary outcome of the study was the change in NFS, a validated non-invasive marker of hepatic fibrosis. The significant reduction in NFS observed with saroglitazar shows its potential antifibrotic benefit in patients with T2DM-associated MASLD. Secondary outcomes, including CAP and LSM, were evaluated to support the fibrosis findings and provide complementary structural assessment.

Saroglitazar led to a reduction in liver stiffness by 1.1 kPa over 12 months. These findings align with the study by Goyal et al., where a significant reduction in liver stiffness was observed from 8.4 to 7.5 kPa (p = 0.0261) over 24 weeks, predominantly in patients with F2/F3 fibrosis [14]. Similarly, Chaudhuri et al. reported a 22% reduction in liver stiffness (8.5 to 6.5 kPa, p<0.001) following saroglitazar therapy [15]. The differences in significance may be attributed to variations in baseline liver stiffness and population characteristics. The dual PPARα/γ activation by saroglitazar is likely responsible for the attenuation of hepatic fibrosis by promoting fatty acid oxidation, reducing hepatocellular inflammation, and decreasing hepatic lipid deposition. A statistically significant reduction in NFS was noted with saroglitazar (-0.57 points, p = 0.026), indicating improvements in both hepatic fibrosis and associated metabolic burden. Regarding hepatic steatosis, saroglitazar achieved an 18.6 dB/m reduction in CAP score over 12 months, approaching statistical significance (p = 0.059). This aligns with the findings of Goyal et al., who observed a decrease from 335 to 256 dB/m (p = 0.0076), and Chaudhuri et al., who showed a 14% decrease (328 to 287 dB/m, p<0.001) [14,15].

These improvements in hepatic steatosis are consistent with the mechanism of PPARα activation, which enhances β-oxidation of fatty acids and reduces hepatic triglyceride accumulation. The variation in the magnitude of CAP score reduction across studies may reflect differences in baseline steatosis severity and patient adherence to lifestyle interventions. A key methodological consideration of the present study is the timing of assessment of hepatic steatosis and fibrosis. Radiological parameters (CAP and LSM) and NFS were evaluated at baseline and at 12 months, rather than at interim three- or six-month time points. Structural changes in liver fat content and fibrosis in MASLD typically evolve gradually, and short-term assessments may not reliably capture clinically meaningful changes, particularly in real-world observational settings. Interim follow-up visits were therefore focused on metabolic parameters and safety monitoring, while fibrosis assessment was reserved for longer-term evaluation to reduce biological variability and measurement noise.

Treatment with saroglitazar demonstrated a significant reduction in HbA1c levels by 0.81% (p<0.0001), compared to a modest reduction of 0.31% with pioglitazone. This finding is consistent with prior studies, including that of Goyal et al., who reported a decrease in HbA1c from 7.2% to 6.3% (p = 0.0001) with saroglitazar, and Roy et al., who noted a mean HbA1c reduction of 0.9% (p<0.0001)[14,16]. Pioglitazone has shown variable glycemic effects in different studies. Goldberg et al. reported a reduction of 0.7% in HbA1c with pioglitazone at a dose of 30 mg/day over six months, while Cho et al. demonstrated a 0.81% reduction at 30 mg/day [17]. The comparatively lower improvement observed with pioglitazone in the present study may be due to the lower 15 mg dose employed. The superior glycemic control achieved with saroglitazar can be attributed to its dual PPAR activation, which not only enhances peripheral insulin sensitivity but also improves hepatic glucose metabolism.

In terms of lipid profile, both saroglitazar and pioglitazone led to mild reductions in total cholesterol, LDL cholesterol, and triglycerides; however, these changes were not statistically significant. In contrast, Chaudhuri et al. reported a 26% reduction in LDL cholesterol, 41% reduction in triglycerides, and 24% reduction in total cholesterol with saroglitazar (all p<0.001)[15]. Goyal et al. similarly observed approximately 50% reduction in triglycerides and a 15 mg/dL reduction in LDL cholesterol (p<0.01). Roy et al. documented a triglyceride reduction of ~96 mg/dL (p<0.0001) and an HDL increase of ~4.4 mg/dL (p = 0.0007)[14]. The modest lipid improvements in the current study could be related to differences in baseline lipid levels, widespread statin use among participants, and dietary variations. Saroglitazar’s PPARα activation is mechanistically linked to increased lipid oxidation and enhanced clearance of triglyceride-rich lipoproteins, supporting its role in improving dyslipidemia.

Saroglitazar also led to a significant reduction in BMI by 3.32 kg/m² (p = 0.006) over 12 months, whereas pioglitazone use was associated with no significant change in BMI. Weight loss is a recognized therapeutic target in MASLD, associated with improvements in steatosis, inflammation, and fibrosis. Previous studies with saroglitazar, including those by Goyal et al. and Roy et al., have reported either modest reductions or stabilization of body weight [14,16]. In contrast, pioglitazone use has often been associated with weight gain, as noted by Cho et al., where a mean increase of 2.61 kg was observed with 30 mg/day dosing (p<0.001), and Goldberg et al., who reported a 2.0 kg increase (p = 0.164) [18]. The weight gain might be due to pioglitazone’s PPARγ-driven adipogenesis.

Although saroglitazar demonstrated a significant reduction in BMI over 12 months, this finding should be interpreted with caution. Saroglitazar is generally considered a weight-neutral agent, and the observed BMI decline may represent a combined effect of the structured lifestyle advice routinely provided to all participants, optimization of background antidiabetic therapy, and individual variations in diet and physical activity. Importantly, lifestyle adherence was not routinely monitored or documented in this real-world study, limiting the ability to attribute weight reduction exclusively to pharmacologic action. This limitation is consistent with previous MASLD studies, which have shown that the magnitude of weight change largely depends on adherence to lifestyle interventions. Future randomized controlled trials with standardized lifestyle protocols or objective activity monitoring are needed to clarify drug-specific effects on body weight.

These findings suggest that saroglitazar may offer a promising therapeutic option for patients with T2DM and MASLD, providing improvements in hepatic fibrosis, steatosis, glycemic control, and body weight without significant safety concerns. The comparative design and modest sample size may limit the generalizability of the findings. Baseline differences in disease severity between the two treatment groups represent an important limitation, as they preclude a fully validated inter-group comparison. Furthermore, lifestyle interventions, which could have influenced outcomes, were not strictly controlled. Larger, randomized controlled trials with histopathological endpoints are warranted to validate these results and further elucidate the long-term benefits of saroglitazar in this population.

## Conclusions

In this prospective, comparative study, saroglitazar demonstrated greater efficacy than pioglitazone in improving hepatic fibrosis, steatosis, glycemic control, and BMI in patients with T2DM and NAFLD over 12 months. Both agents were well tolerated with no major adverse events. Although changes in lipid profile and insulin resistance, assessed using the TyG index, were modest, saroglitazar’s dual PPARα/γ activation provides a mechanistic advantage by targeting multiple metabolic pathways. These findings support the potential role of saroglitazar in managing T2DM-associated MASLD, although larger, randomized studies are needed to confirm its long-term benefits.
